# Informational masking influences segmental and suprasegmental speech categorization

**DOI:** 10.3758/s13423-023-02364-5

**Published:** 2023-09-01

**Authors:** A. E. Symons, L. L. Holt, A. T. Tierney

**Affiliations:** 1 Department of Psychological Sciences, Birkbeck, University of London, London, UK; 2 Department of Psychology and Neuroscience Institute, Carnegie Mellon University, 500 Forbes Avenue, Pittsburgh, PA USA

**Keywords:** Speech perception, Categorization, Selective attention, Individual differences

## Abstract

Auditory categorization requires listeners to integrate acoustic information from multiple dimensions. Attentional theories suggest that acoustic dimensions that are informative attract attention and therefore receive greater perceptual weight during categorization. However, the acoustic environment is often noisy, with multiple sound sources competing for listeners’ attention. Amid these adverse conditions, attentional theories predict that listeners will distribute attention more evenly across multiple dimensions. Here we test this prediction using an informational masking paradigm. In two experiments, listeners completed suprasegmental (focus) and segmental (voicing) speech categorization tasks in quiet or in the presence of competing speech. In both experiments, the target speech consisted of short words or phrases that varied in the extent to which fundamental frequency (F0) and durational information signalled category identity. To isolate effects of informational masking, target and competing speech were presented in opposite ears. Across both experiments, there was substantial individual variability in the relative weighting of the two dimensions. These individual differences were consistent across listening conditions, suggesting that they reflect stable perceptual strategies. Consistent with attentional theories of auditory categorization, listeners who relied on a single primary dimension in quiet shifted towards integrating across multiple dimensions in the presence of competing speech. These findings demonstrate that listeners make greater use of the redundancy present in speech when attentional resources are limited.

**Supplementary Information:**

The online version contains supplementary material available at 10.3758/s13423-023-02364-5.

Speech categorization presents a model of how learners weight multiple sources of information in perceptual decision-making. When categorizing speech sounds, listeners must learn to map variations along multiple acoustic dimensions onto discrete categories. Under ideal listening conditions, these dimensions are not given equal priority: acoustic dimensions carry different *perceptual weight*. For example, evidence suggests that voice onset time (VOT; the time that elapses between the release of the consonant and onset of vocal fold vibration) is the primary cue to voicing in English, and is the dimension on which native English speakers most heavily rely (Keating, 1984; Lisker & Abramson, 1964). By contrast, the fundamental frequency (F0) of the following vowel provides a less reliable cue to voicing and receives less perceptual weight during categorization. This differential weighting of acoustic dimensions has also been observed across suprasegmental features including syllable stress (Fear et al., 1995; Mattys, 2000), phrase boundary location (de Pijper & Sanderman, 1994; Jasmin et al., 2021; Streeter, 1978), and linguistic focus (Breen et al., 2010; Jasmin et al., 2019).

According to computational models of speech categorization, listeners weight different acoustic dimensions according to the reliability with which these dimensions distinguish between categories (Toscano & McMurray, 2010). However, the reliability of acoustic dimensions varies depending on both the listener and their environment. With few exceptions (e.g., Holt et al., 2018; Winn et al., 2013; Wu & Holt, 2022), studies of perceptual weighting have been carried out under ideal laboratory conditions. But speech perception in natural environments involves noise, competing talkers and other factors that might impact how consistently sensory input signals category membership. Listeners therefore need to adjust their dimensional weights to cope with these more challenging listening conditions.

There are multiple ways in which natural listening environments might impact perceptual weighting in categorization. For example, noise or competing speech from another talker may produce energetic masking, which occurs when spectrotemporal overlap between the target and competing speech masks acoustic information in the target speech. This type of energetic masking can alter listeners’ dimensional weights in cases where noise obscures the availability of particular dimensions. For example, F0 carries greater perceptual weight than VOT in noise, opposite the pattern observed in quiet (Holt et al. 2018; Winn et al., 2013; Wu & Holt, 2022). Informational masking (Brungart, 2001; Brungart et al., 2001; Kidd et al., 2008), which can be defined as interference not driven by spectrotemporal overlap between the target and competing signals, might also contribute. One reason that informational masking can occur is due to the attentional demands required to focus on the target talker in the presence of competing speech (Shinn-Cunningham, 2008).

Theoretical accounts propose a role for selective attention in speech categorization (Francis et al., 2000; Francis & Nusbaum, 2002; Gordon et al., 1993; Heald & Nusbaum, 2014; Holt et al., 2018). According to these accounts, listeners direct attention towards acoustic dimensions that are most informative for categorization. For example, during voicing categorization, most English listeners direct processing resources towards the primary dimension (VOT) relative to the secondary dimension (F0; Gordon et al., 1993). Under this view, the primary dimension is the target of attention. Conditions that limit attentional resources (e.g., noise, talker variability, other concurrent task demands) will lead to a down-weighting of the primary dimension as listeners are less able to continuously fix their attention on a single dimension, instead distributing processing more evenly across dimensions (Francis et al., 2008). Two prior studies of segmental categorization provide initial support for this possibility. Under conditions of high compared with low cognitive load, listeners down-weight the primary dimension during segmental categorization (Gordon et al., 1993; Kong & Lee, 2018). Moreover, listeners who strongly weight a single primary dimension under conditions of low cognitive load show a greater down-weighting of that dimension when engaged in a cognitively demanding task (Kong & Lee, 2018). These findings suggest that primary dimensions may be the target of attention and that decreasing the available cognitive resources may limit the extent of primary weighting. However, these studies manipulated attention using a dual-task paradigm in which listeners answered a math question in between hearing the speech stimulus and making a categorization response. This type of dual-task manipulation of cognitive load is less directly related to real-world listening.

If selective attention plays a central role in speech categorization in real-world listening, we predict that perceptual weighting will be influenced by listening to speech in the context of a competing talker. We test this prediction across perception of segmental (voicing) and suprasegmental (linguistic focus) categories. Although no prior study has investigated the effects of informational masking on perceptual weighting, based on prior work using dual-task paradigms (Gordon et al., 1993; Kong & Lee, 2018), we predicted that the presence of competing speech would decrease listeners’ weighting of the primary dimension relative to the quiet listening conditions under which most studies of perceptual weighting have been conducted. Moreover, if selective attention is a general mechanism that underpins auditory categorization, we predicted that informational masking would lead to a shift in primary weighting across both segmental and suprasegmental categorization tasks.

However, these predictions refer to the primary dimension for a given categorization task, which may differ across listeners. Even in quiet listening conditions, individuals differ in the weight they assign to different dimensions, with some listeners relying on a single primary dimension and others integrating across multiple dimensions (Clayards, 2018; Kapnoula et al., 2017, 2021; Kapnoula & McMurray, 2021; Kim et al., 2018; Kong & Edwards, 2016; Symons & Tierney, 2023). These individual differences are stable across time (Idemaru et al., 2012; Kim et al., 2018). Based on these findings, we predicted that the effect of informational masking on perceptual weighting would vary across listeners. Specifically, we predicted that listeners who strongly weight a single primary dimension in quiet would show a greater shift in their dimensional weights in the presence of competing speech.

In two experiments, we investigated the effect of informational masking on suprasegmental and segmental categorization. Listeners in each experiment completed suprasegmental (focus) and segmental (voicing) categorization tasks in which they heard short phrases that varied orthogonally in pitch (F0) and duration dimensions. Each task was completed in quiet and in the presence of competing speech. To isolate effects of informational masking (and avoid effects of energetic masking), the target and competing speech were always presented to opposite ears. Based on attentional theories of speech categorization, we predicted that informational masking would leave listeners less able to selectively attend to the relevant acoustic dimensions, and therefore lead to a down-weighting of the primary dimension in both suprasegmental and segmental categorization tasks. Moreover, we predicted that listeners who strongly weighted a single primary dimension in quiet would show a greater down-weighting of that dimension under conditions of informational masking.

## Methods

### Participants

In Experiment 1 (suprasegmental categorization), 93 native English speakers between the ages of 18 and 40 years (mean age = 29.84 years, *SD* = 5.76, 78 female, 15 male) were recruited from the Prolific online recruitment service (prolific.co). Since no previous study has tested the effects of informational masking on suprasegmental speech categorization, this sample size was chosen based on previous research showing significant effects of pitch and duration on focus categorization in quiet listening conditions (Symons & Tierney, 2023). To ensure that participants were wearing headphones throughout the experiment, all participants completed a headphone screening test (Milne et al., 2021). Only participants who reached a threshold of 4/6 on the screening test were included.^1^ To ensure online participant engagement with the categorization task, only data from participants for whom there was a significant relationship (*p* < .01; Jasmin et al., 2021) between at least one of the stimulus dimensions and categorization responses (in both clear and competing speech conditions) were included in the final analysis. The final sample consisted of 68 participants (mean age = 29.97 years, *SD* = 5.66, 54 female, 14 male).

In Experiment 2 (segmental categorization), 35 native English speakers between the ages of 18 and 40 years (mean age = 24.71 years, *SD* = 6.56, 22 female, 11 male, 2 nonbinary) were recruited from the university participant pool at Carnegie Mellon (*n* = 9) and from Prolific (*n* = 26). As with Experiment 1, only participants who reached a threshold of 4/6 on the headphone screening test and who showed a significant relationship between at least one stimulus dimension and categorization responses were included. The final sample consisted of 29 participants (mean age = 25.45 years, *SD* = 6.87, 19 female, 9 male, 1 nonbinary). A power analysis based on the results of Experiment 1 suggested that this sample size provided 87% power to detect a correlation with an *r* value of 0.51.

For all participants recruited through Prolific, an automated screening procedure accepted only participants who reported speaking English as a native language and their age as between 18 and 40 years. This was confirmed by responses to an additional questionnaire. There were no geographical restrictions on participation. The experiment was conducted via the online experiment platform Gorilla Experiment Builder (Anwyl-Irvine et al., 2020). Automated procedures ensured that participants completed the experiment on a desktop or laptop using Google Chrome browser. All participants were asked to wear headphones throughout the experiment.

All experimental procedures were approved by the Ethics Committee in the Department of Psychological Sciences at Birkbeck, University of London, and by the University Institutional Review Board at Carnegie Mellon.

### Stimuli

The focus stimuli were obtained from the Multidimensional Battery of Prosody Perception (Jasmin et al., 2020). These stimuli were derived from recordings made by a male southern British English-speaking voice actor reading aloud two different sentences (capitalization indicating contrastive focus): “Dave likes to STUDY music, but he doesn’t like to PLAY music” and “Dave likes to study MUSIC, but he doesn’t like to study HISTORY.” The first five words from each recording were extracted to obtain two versions of the same phrase (“Dave likes to study music”) that differed in the location of linguistic focus (“study” versus “music”). The voice morphing software STRAIGHT (Kawahara & Irino, 2005) was used to create stimuli that varied in the extent to which changes in F0 or duration cued the focused word. Using the standard procedure in STRAIGHT, F0, aperiodicity, and filter characteristics of each version of the phrase were analyzed and synthesized into two morphing substrates which represent the speech signal decomposed into F0, aperiodic components, and filter characteristics. The morphing substrates were manually time-aligned by marking corresponding anchor points in each recording. F0 and duration were morphed along five levels that reflect the relative contribution of each original recording to the morphed stimulus: 1 (100% “STUDY music,” 0% “study MUSIC”), 2 (75% “STUDY music,” 25% “study MUSIC”), 3 (50% “STUDY music,” 50% “study MUSIC”), 4 (25% “STUDY music,” 75% “study MUSIC”), 5 (0% “STUDY music,” 100% “study MUSIC”). This resulted in 25 unique focus stimuli (mean duration = 1.55 seconds, *SD* = 0.09), one for each combination of F0 and duration levels.

The voicing stimuli were derived from natural productions of *beer* and *pier* from a female native American English speaker (LH), chosen for their similarity in duration and fundamental frequency (F0) contour. Following the approach of McMurray and Aslin (2005), VOT was manipulated from 5 to 25 ms in 5-ms steps using Praat (Boersma & Weenink, 2019). Although a narrower range of VOT values compared to some previous research (McMurray & Aslin, 2005), pilot testing ensured that this range allowed us to detect a relationship between each stimulus dimension and categorization responses. Next, vowel onset F0 was manipulated from 220 to 300 Hz in 20-Hz step sizes using Praat (Boersma & Weenink, 2019). The F0 contour decreased quadratically to 150 Hz at stimulus offset. The resulting stimulus space consisted of 25 unique voicing stimuli (mean duration = 0.469 seconds, *SD* = 0.01), one for each combination of voice onset time and fundamental frequency (Idemaru & Holt, 2011), normalized to have the same root-mean-squared amplitude.

The competing speech stimuli consisted of English speech productions produced by a male American English speaker for a coordinate response measure (CRM) paradigm (Bolia et al., 2000; Smith et al., 2022). The original recordings consisted of the sentence “Ready [call sign], go to [color] [number] now.” There were seven possible call signs (arrow, baron, eagle, hopper, laker, ringo, tiger), three colors (red, green, blue), and seven numbers (1, 2, 3, 4, 5, 6, 8). These recordings were shortened in length at the zero crossings (mean duration = 2.02 seconds, *SD* = 0.12) and RMS matched (mean dB = −13.98). This resulted in 147 unique competing speech stimuli.

In the competing speech condition, each target stimulus (25 voicing stimuli, 25 focus stimuli) was paired with 10 different competing speech stimuli, selected at random. The target and competing speech stimuli were mixed into a single sound file with the target and competing speech stimuli presented in opposite ears to prevent energetic masking. On half of the trials, the target stimulus was presented in the left ear, and in the other half of the trials, the target stimulus was presented in the right ear. The target stimulus could occur at any point from 200-ms after the onset of the competing speech. Because of the temporal delay between competing and target speech, listeners knew which ear to attend to and which to ignore upon the onset of the competing speech.

In the clear speech condition, a 100-ms 1-kHz tone was inserted instead of the competing speech stimuli to control for temporal cueing effects. The temporal delay between the onset of the tone and the target stimulus speech was matched to the competing speech condition. Each of the stimuli in the clear speech condition was presented 10 times so that the number of trials was equivalent between clear and competing speech conditions (500 trials per experiment).^2^ The 1-kHz tone and competing speech stimuli were matched in loudness to the target stimulus using the stationaryLoudness function implemented in MATLAB (Swift & Gee, 2019).

### Procedure

The procedure for Experiments 1 and 2 was identical. Upon signing up to the study, participants were sent a link to the experiment. After providing informed consent, participants completed a short demographic questionnaire in which they provided information about their age, gender, language background (native language and other languages spoken), and musical experience (years of training, age at which training began, instruments on which they were instructed).

To check whether participants were wearing headphones, participants completed a short headphone screening test where a faint tone can be detected in noise only when the stimuli are presented dichotically (Milne et al., 2021). Participants who failed to achieve a score of at least 4/6 on the headphone screening test were excluded from analysis. To ensure that this decision did not bias our results, we include an analysis with a 6/6 criterion in the Supplementary Materials.

Prior to the categorization tasks, participants were presented with a set of instructions alongside examples of the target stimuli from two of the corners of the stimulus space where both stimulus dimensions unambiguously signaled category identity. Participants were asked to play each example 3 times before proceeding to the practice trials. There were two practice trials, consisting of the same stimuli as the examples. During the practice trials, participants listened to a single stimulus (with no visual information on the screen) and categorized the stimulus by pressing one of two buttons that appeared on-screen following stimulus presentation. When the response was incorrect, the word “Nope …” appeared on the screen. The feedback remained on the screen until participants clicked a button to move onto the next trial. The trial structure of the main tasks was identical to the practice except that feedback was no longer provided.

During the main task, clear and competing speech conditions were presented in separate blocks, with the order of blocks counterbalanced across participants. For each condition, participants were presented each of the 250 stimuli, one at a time in randomized order, and responded by pressing an on-screen button to indicate whether the word spoken by the target speaker resembled *“STUDY music”* or *“study MUSIC”* in the focus task or *“beer”* or *“pier”* in the voicing task. No feedback was provided on the main task and self-paced breaks were provided every 50 trials.

### Data analysis

#### Group analysis

To test the effect of informational masking on perceptual weighting, we constructed mixed effects logistic regression models for focus and voicing categorization tasks using the *lme4* package (Bates et al., 2015) in R (R Core Team, 2022). This analysis allowed us to test the extent to which categorization responses were influenced by each dimension in clear versus competing speech conditions.

For the focus model, the dependent variable was response on each trial (1 = study MUSIC, 0 = STUDY music). Predictors included condition (clear speech, competing speech) as a categorical variable, F0 level (1–5) and duration level (1–5) as continuous variables, and the interaction between condition and each dimension. Item and participant were included as random intercepts with by-participant random slopes included for condition, F0 level, duration level, and the interaction between condition and each dimension. We predicted an interaction between condition and F0 level, reflecting a decreased influence of F0 on categorization responses in the presence of competing speech.

For the voicing model, the dependent variable was response on each trial (1 = pier, 0 = beer). Predictors included condition (clear speech, competing speech) as a categorical variable, VOT level (1–5) and F0 level (1–5) as continuous variables, and the interaction between condition and each dimension. Item and participant were included as random intercepts with by-participant random slopes for condition, VOT level, F0 level, and the interaction between condition and each dimension. We predicted an interaction between condition and VOT level, reflecting a decreased influence of VOT on categorization responses in the presence of competing speech.

For both models, categorical predictors were centered (−0.5, 0.5) and continuous predictors were standardized by centering and dividing by 2 standard deviations using the *rescale()* function in the arm package in R (Gelman, 2008).

#### Individual differences

To examine individual differences in the effect of informational masking on dimensional weights, a logistic regression model was constructed for each participant. For Experiment 1, F0 and duration (levels 1–5) were continuous predictors and response on each trial was the outcome variable. For Experiment 2, VOT and F0 (levels 1–5) were continuous predictors and categorization response on each trial was the outcome variable. Following Holt and Lotto (2006), regression coefficients were extracted and normalized such that the absolute values of the coefficients summed to 1, providing a measure of the relative weighting of each dimension during categorization. For the focus task, normalized F0 weights were computed by dividing absolute value of F0 coefficients by the sum of the absolute value of F0 and duration coefficients. For the voicing task, normalized VOT weights were computed by dividing the absolute value of VOT coefficients by the sum of the absolute value of VOT and F0 coefficients. We then used Spearman’s correlations to test the consistency of listeners’ normalized dimensional weights across clear and competing speech conditions.

To test the prediction that listeners’ degree of primary weighting (irrespective of which dimension was primary for the individual) would shift in the presence of competing speech, we computed an index of listeners’ *primary cue weight*. In the focus task, primary cue weights for individuals with normalized F0 weights less than 0.5 (indicating that duration was primary) were defined as 1 minus the normalized F0 weight. Primary cue weights for individuals with normalized F0 weights greater than 0.5, were equivalent to the normalized F0 weight. Similarly, in the voicing task, primary cue weights for individuals with normalized VOT weights less than 0.5 (indicating that F0 was primary) were defined as 1 minus the normalized VOT weight. Primary cue weights for individuals with normalized VOT weights greater than 0.5, were equivalent to the normalized VOT weight. This resulted in a measure ranging from 0.5 to 1, with 1 indicating strong weighting of a single primary dimension and 0.5 indicating an equal weighting of the two dimensions. Spearman’s correlations were used to test whether listeners who placed greater weight on a given dimension in clear speech would show a greater down-weighting of that dimension in the presence of competing speech. To do this, we computed an index of listeners’ *primary weighting shift*, which was defined as the difference in primary cue weights between clear and competing speech conditions. Positive values represent greater primary weighting in clear compared to competing speech conditions (indicative of a down-weighting of the primary dimension in competing speech conditions) while negative values represent less primary weighting in the clear compared to competing speech conditions.

## Results

### Focus categorization

**Fig. 1:**
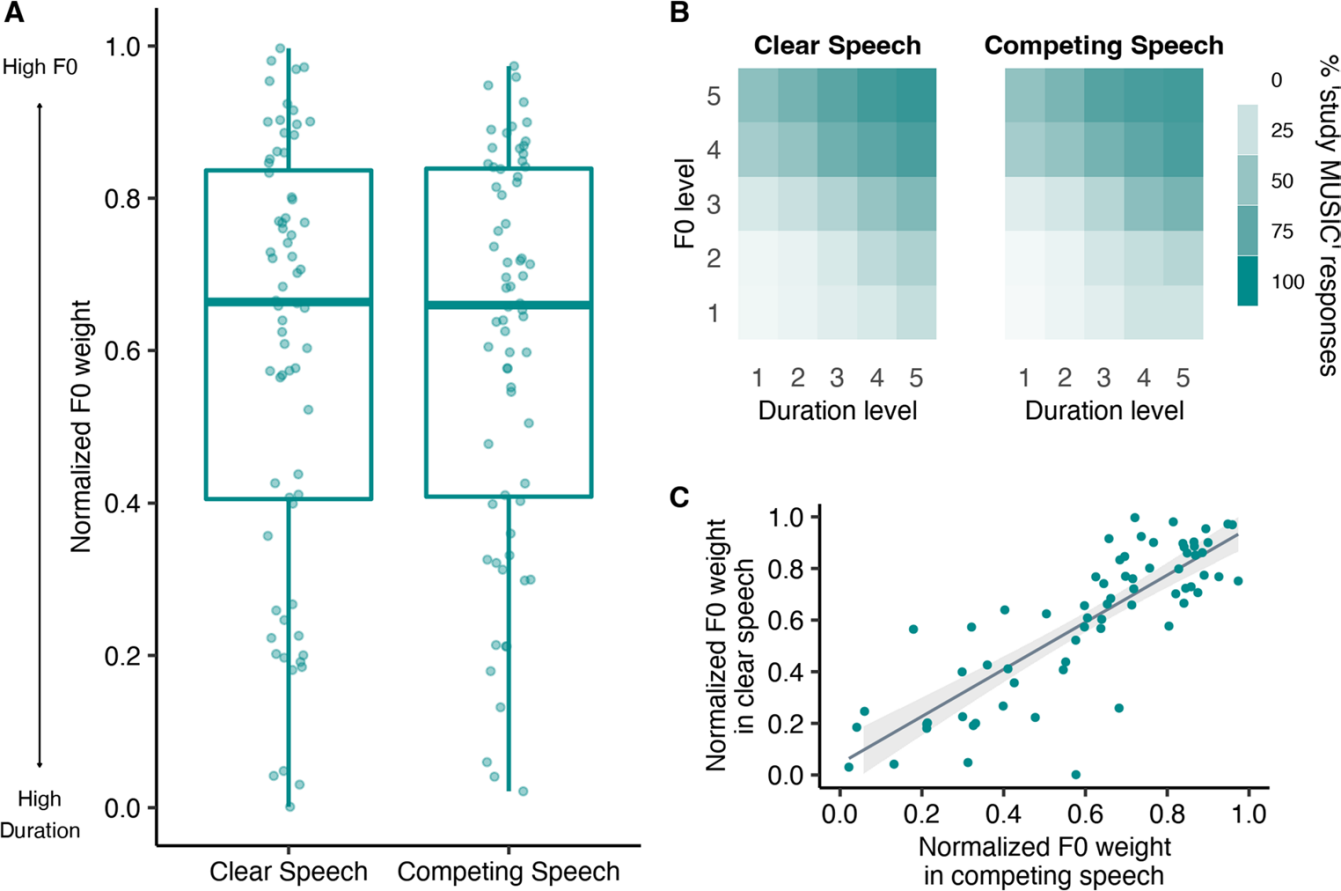
**A** Boxplots showing normalized F0 weights in the clear and competing speech conditions. **B** Heatmaps showing the percentage of ‘study MUSIC’ responses in clear and competing speech conditions, with darker colors indicating a higher proportion of ‘study MUSIC’ responses and lighter colors indicating a higher proportion of ‘STUDY music’ responses. **C** Relationship between normalized cue weights in the clear and competing speech conditions. (Color figure online)

Figure 1 shows normalized dimensional weights in the clear (median = 0.66) and competing (median = 0.66) speech conditions along with heatmaps displaying the average percentage of ‘study MUSIC’ responses for each level of F0 and duration. Visual inspection of the data suggests that both F0 and duration influenced categorization responses. However, there was substantial variability across listeners as to which dimension was primary. Correlational analyses showed consistency in listeners’ perceptual weighting strategies across clear and competing speech conditions (rho = 0.83, *p* < .001; Fig.1C).

Results of the mixed effects model are summarized in Table 1. Both F0 (β = 3.19, *z* = 11.62, *p* < .001) and duration level (β = 1.63, *z* = 11.92, *p* < .001) influenced listeners’ focus categorization decisions. However, listening condition did not influence perceptual reliance on either dimension.

**Table 1:** Summary of fixed effects in the mixed-effects logistic regression models for Experiment 1 (focus categorization)

	Estimate	*SE*	*z* value	*p* value
(Intercept)	−0.54	0.09	−6.24	<.001
Condition	−0.06	−0.06	−0.98	.33
F0	3.19	0.27	11.62	<.001
Duration	1.63	0.14	11.92	<.001
Condition × F0	−0.10	0.12	−0.81	.42
Condition × Duration	0.01	0.11	0.08	.94

The reference level for condition is the clear speech condition

**Fig. 2:**
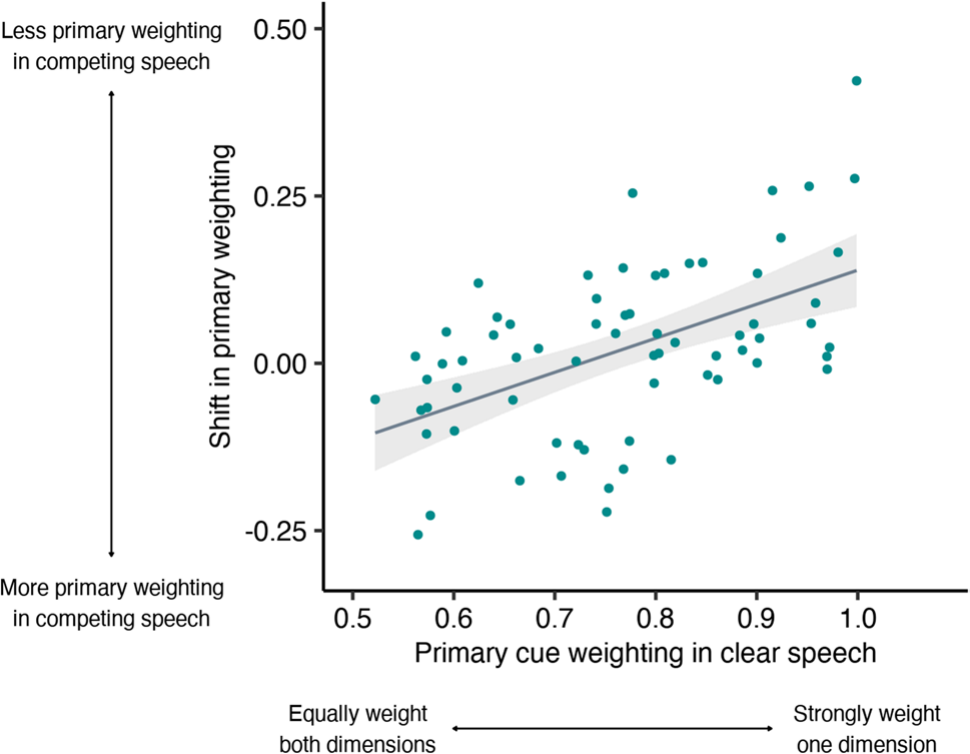
Relationship between primary weighting in clear speech and listeners’ shift in primary weighting in the presence of competing speech. Listeners who strongly weighted a single primary dimension in quiet shifted towards integrating across dimensions in the presence of competing speech (*p* <. 0.001)

We then tested the hypothesis that listeners who more strongly weight a given dimension in clear speech under optimal listening conditions will show larger shifts in weighting of that dimension when attentional resources are limited. As shown in Fig. 2, listeners who tended to rely heavily on a single dimension in quiet (rather than weighting the two dimensions more equally) showed a greater shift towards integrating across multiple dimensions in the presence of competing speech (rho = 0.51, *p* < .001).

#### Summary

During focus categorization, there are consistent individual differences in perceptual weighting across listening conditions. While some listeners strongly weight a single primary dimension, others more equally weight the two dimensions. However, when attentional resources are limited due to the presence of a competing talker, listeners converge towards integrating across multiple dimensions, with individuals who strongly weight a single dimension in quiet showing the greatest shift.

### Voicing categorization

**Fig. 3:**
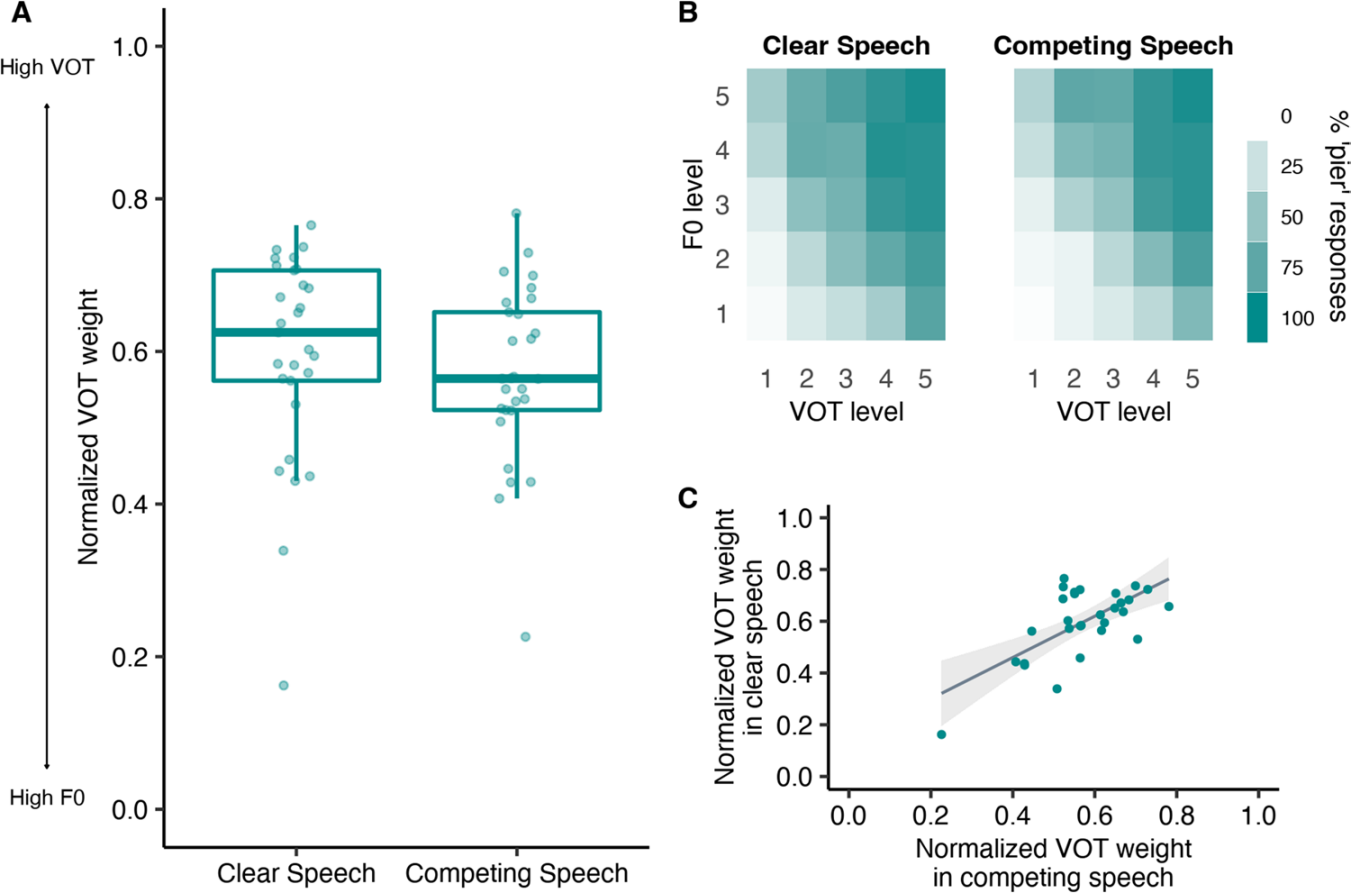
**A** Boxplots showing normalized VOT weights in the clear and competing speech conditions. **B** Heatmaps showing the average percentage of ‘pier’ responses with darker colors indicating a higher proportion of ‘pier’ responses and lighter colors indicating a higher proportion of ‘beer’ responses. **C** Relationship between normalized dimensional weights across clear and competing speech conditions. (Colour figure online)

Figure 3 shows normalized dimensional weights in the clear (median = 0.63) and competing speech (median = 0.56) conditions along with heatmaps displaying the average percentage of ‘pier’ responses for each level of VOT and F0. Visual inspection of the data suggests that both VOT and F0 influenced categorization responses, but with variability across listeners as to which dimension was primary. Correlational analyses showed a significant relationship between normalized VOT weights across conditions (rho = 0.43, *p* = .02), suggesting consistency in dimensional weights across clear and competing speech conditions.

Results of the mixed effects logistic regression are summarized in Table 2. As expected, both VOT (β = 3.48, *z* = 13.07, *p* < .001) and F0 level (β = 2.32, *z* = 10.59, *p* < .001) influenced categorization responses. Overall, the proportion of ‘pier’ responses was lower in the clear compared to competing speech conditions (β = −0.56, *z* = −4.82, *p* < .001). An interaction between condition and F0 level (β = 0.53, *z* = 2.56, *p* = .01) showed that F0 had a greater influence on categorization responses in competing compared to clear speech conditions.

**Table 2:** Summary of fixed effects in the mixed-effects logistic regression models for Experiment 2 (voicing categorization)

	Estimate	*SE*	*z* value	*p* value
(Intercept)	0.78	0.17	4.51	< 0.001
Condition	-0.56	0.12	-4.82	< 0.001
VOT	3.48	0.27	13.07	< 0.001
F0	2.32	0.22	10.59	< 0.001
Condition x VOT	0.08	0.26	0.29	0.77
Condition x F0	0.53	0.21	2.56	0.01

The reference level for condition is the clear speech condition

**Fig. 4:**
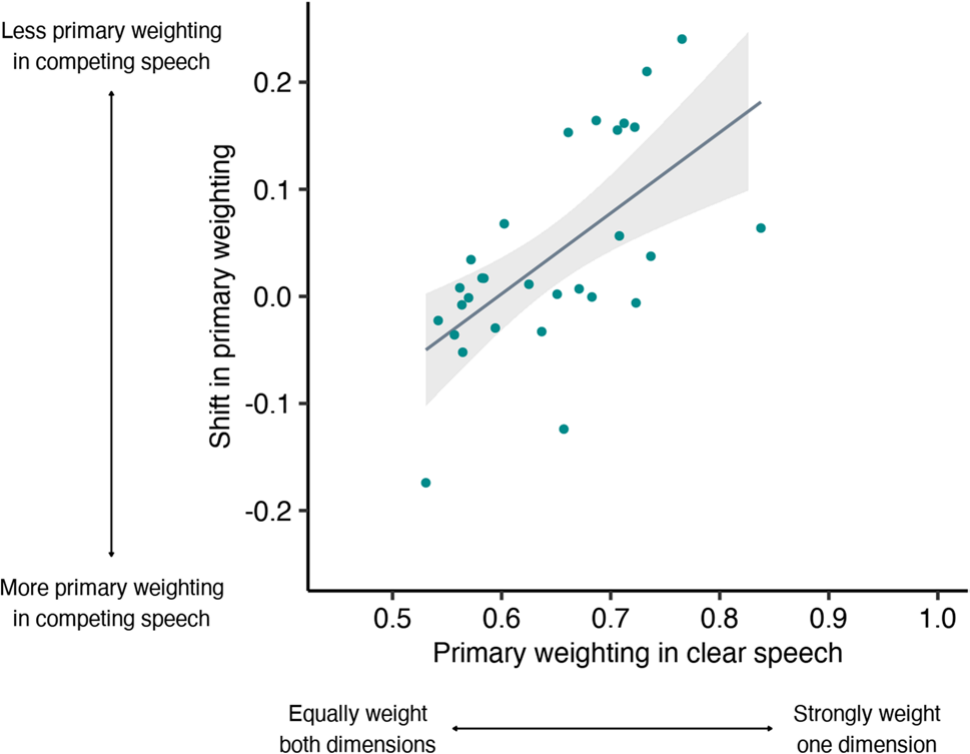
Relationship between primary weighting in clear speech and listeners’ shift in primary weighting in the presence of competing speech during voicing categorization. Listeners who strongly weighted a single primary dimension in quiet showed a greater shift towards integrating across dimensions (less weighting of the primary dimension) in the presence of competing speech (*p* < .001)

As shown in Fig. 4, listeners who tended to rely on a single primary dimension in clear speech shifted towards integrating across multiple dimensions in the presence of competing speech (rho = 0.67, *p* < .001).

#### Summary

During voicing categorization, listeners showed consistent perceptual weighting strategies across listening conditions. At the group level, the presence of competing speech led to an upweighting of F0 (the secondary dimension on average), reflecting a shift toward integrating across dimensions. However, the magnitude of this shift was predicted by the extent to which individual listeners relied on a single dimension in quiet.

## Discussion

In two experiments, we show that selective attention influences perceptual weighting during speech categorization. Across both segmental and suprasegmental categorization tasks, listeners shifted their perceptual weights when attentional resources were limited by the presence of competing speech. However, the magnitude of this shift was predicted by listeners’ primary weighting in quiet; listeners who relied on a single dimension in quiet shifted towards integrating across multiple dimensions in competing talker environments.

Based on attentional theories of perceptual weighting (Francis et al., 2000; Francis & Nusbaum, 2002; Gordon et al., 1993; Heald & Nusbaum, 2014; Holt et al., 2018), we predicted that listeners would down-weight the primary dimension in the presence of competing speech. At the group level, our findings were only partially consistent with this prediction; listeners in the voicing categorization task upweighted the secondary dimension (F0) in competing compared to clear speech conditions. Why this attentional manipulation led to an upweighting of the secondary dimension rather than a down-weighting of the primary dimension (as in Gordon et al., 1993; Kong & Lee, 2018) is unclear. Upweighting of the secondary dimension has been observed in studies of energetic masking (Winn et al., 2013), but the presentation of target and competing speech in opposite ears combined with the headphone screening test make this explanation unlikely. Moreover, this effect was not observed in the focus task, potentially because neither dimension could be considered ‘primary’ on average based on the variability in the data (see also Symons & Tierney, 2023). However, when accounting for individual differences in primary weighting under quiet listening conditions, our results provide clear support for theories that propose a role for selective attention in auditory categorization. As suggested by Francis et al. (2008), noisy distracting listening conditions that limit attentional resources may lead to a “more even distribution of resources” across dimensions. Our findings are in line with this idea; individuals who direct attention towards a single primary dimension in quiet redistribute their attention more evenly in the presence of competing speech while individuals who already show an even distribution of attentional resources in quiet show less of a shift. This pattern was observed across both segmental and suprasegmental categorization tasks, each with a different primary and secondary dimension, suggesting that selective attention represents a general mechanism underpinning speech categorization.

Speech is a redundant signal in which information about speech categories is conveyed via multiple acoustic dimensions (Winter, 2014). According to weighting-by-reliability models of speech categorization, listeners make use of multiple acoustic dimensions, but place greater perceptual weight on the acoustic dimensions that are most reliable based on the distributional statistics of the input (Toscano & McMurray, 2010). Consistent with these models, in quiet, listeners tend to place greater weight on certain primary dimensions, such as VOT for voicing in English, compared to secondary dimensions such as F0 (Keating, 1984; Lisker & Abramson, 1964). This perceptual weighting strategy may be optimal when a single dimension can provide sufficient evidence to distinguish between categories. However, this strategy may not be optimal under more challenging listening conditions. In the case of informational masking, listeners’ ability to attend toward acoustic information in the target speech may be diminished due to distraction caused by the presence of competing speech. As a result, listeners may converge towards a strategy in which they integrate across multiple dimensions, making greater use of multiple sources of information. Making use of multiple sources of information may be particularly beneficial because different dimensions tend to unfold across different temporal scales; a listener who relies on multiple dimensions can successfully categorize speech even if information carried by the primary dimension was missed due to a lapse of attention because they can still use information from a different dimension at another time point (see Winter, 2014, for a similar argument in the case of energetic masking). However, as our results show, the degree to which informational masking influences perceptual weighting will vary across individuals; those who strongly weight a single dimension under optimal listening conditions show a stronger shift towards integrating across dimensions in the presence of competing speech.

Prior work has shown substantial individual differences in perceptual weighting during segmental (Idemaru et al., 2012; Kapnoula et al., 2017, 2021; Kapnoula & McMurray, 2021; Kim et al., 2018, 2020; Kong & Edwards, 2016) and suprasegmental (Jasmin et al., 2019, 2023; Symons & Tierney, 2023) categorization under quiet listening conditions. In line with this work, we observed consistent individual differences in dimensional weights during segmental and suprasegmental categorization in both listening conditions. However, under conditions of informational masking, listeners shifted towards integrating across multiple dimensions. This implies that individual differences in perceptual weighting may be more pronounced in quiet compared with real-world listening conditions where multiple sound sources compete for listeners’ attention. The present work also suggests a potential refinement of computational models, which assume a single, optimal perceptual weighting strategy based on the distributional statistics of the input (Toscano & McMurray, 2010). Perceptual weighting strategies that deviate from this pattern, such as upweighting of F0 during voicing categorization in older adults (Toscano & Lansing, 2019), are treated as suboptimal. However, in some cases, what appear to be ‘suboptimal’ strategies at the group level may in fact be optimal for the individual based on their unique auditory system (Jasmin et al., 2019). Yet one key question raised by the present study is what leads some listeners to adopt more flexible perceptual weighting strategies across different listening conditions. In line with attentional theories (Francis et al., 2000; Francis & Nusbaum, 2002; Gordon et al., 1993; Heald & Nusbaum, 2014; Holt et al., 2018), one possibility is that the ability to selectively attend to acoustic dimensions predicts the degree to which listeners can flexibly adapt their dimensional weights depending on the listening condition.

These experiments were conducted online, which has a number of advantages over in-lab testing including access to larger and more representative participant samples (Buhrmester et al., 2011; Gosling et al., 2004). Although this meant that we had less control over participants’ acoustic environments, the fact that individual differences in listeners’ dimensional weights correlated across listening conditions suggests that they reflect stable perceptual strategies rather than noise due to an inconsistent acoustic environment. That is, regardless of listening condition, some individuals consistently upweight a single dimension while others integrate across multiple dimensions. Prior work suggests that these individual differences in perceptual weighting may be driven by auditory perceptual ability (Jasmin et al., 2019) as well as auditory experience within (Jasmin et al., 2021) and outside (Symons & Tierney, 2023) the domain of language. Another factor which may have influenced individual differences in the degree of informational masking, particularly for the focus task where speaker accents differed, is accent familiarity. Given that we observed the same pattern of results across tasks, differences in accent familiarity are unlikely to be the main driver of our results but may contribute to unexplained variability in the degree of informational masking in the focus task. Thus, although variability in the participant sample and testing conditions is an inevitable part of online research, we still find stable individual differences in perceptual weighting across listening conditions, and similar effects of informational masking across different tasks.

In conclusion, our findings suggest that the dimensional weights observed under ideal laboratory conditions do not necessarily reflect the way in which listeners integrate information from acoustic dimensions under real-world listening conditions. In naturalistic listening environments, competing acoustic signals can obscure acoustic cues to speech perception while at the same time making it more difficult to control the focus of attention. Adding to this challenge, speech perception often takes place while the listener is engaged in other tasks. Under these real-world listening conditions, it may be more beneficial to use multiple sources of information rather than rely on a single primary dimension. Our findings suggest that this is exactly what listeners do; in the presence of competing speech, listeners who rely on a single dimension in quiet show a stronger tendency to shift towards integrating across multiple dimensions in the presence of competing speech. If research conducted in the lab is to generalize to real-world listening environments, future research needs to include conditions that better approximate the environments in which speech perception occurs in everyday life.

### Supplementary Information

Below is the link to the electronic supplementary material.

Supplementary file1 (DOCX 18 KB)

## Data Availability

Processed data are available online (https://osf.io/9bwj5/). Raw data are available on request from the corresponding author.

## Footnotes

1 To ensure that this choice of threshold did not bias our results, analysis of results with a 6/6 threshold is included in the Supplementary Materials.

2 Due to a programming error, one stimulus per condition was replaced with another in the third block (in both clear and competing speech conditions). This error affected <0.5% of the data. The Supplementary Materials includes an analysis excluding this block from analysis. All results remain the same.
