# Supplementary material for: Informational masking influences segmental and suprasegmental speech categorization
Source: Psychon Bull Rev. 2023 Sep 1;31(2):686–96. doi: 10.3758/s13423-023-02364-5 (PMC11061029; doi:10.3758/s13423-023-02364-5)
Supplement: Supplementary file 1 — Supplementary file1 (DOCX 18 KB) [file 13423_2023_2364_MOESM1_ESM.docx]

**Supplementary Materials**

**Overview**

Due to a programming error, one stimulus (F0 level 1, duration level 1) was repeated while another stimulus (F0 level 2, duration level 1 in the focus task and duration level 2, F0 level 1, in the voicing task) was omitted in the third block of the task for each subject. This error affected 1 trial (/250 trials) per condition per participant (< 0.5% of trials). To ensure that this error did not influence the results, the analysis below removes the third block from analysis. Additionally, we exclude participants who achieved a score of < 6/6 on the headphone screening check to ensure that our choice of threshold did not bias our results. This analysis yielded an identical pattern of results to the analysis reported in the main text. Code to reproduce this analysis is available at: <https://osf.io/9bwj5/>

**Results**

*Focus categorization*

Consistent with the results described in the main text, there was a significant effect of F0 and duration on categorization responses (Table S1), but no effect of condition. Correlational analyses showed stable individual differences in cue weighting across clear and competing speech conditions (rho = 0.785, p < 0.001). Listeners who strongly weighted a single primary dimension in quiet showed a greater shift towards integrating across multiple dimensions in the presence of competing speech (rho = 0.510, p < 0.001).

**Table S1.** Results of mixed effects model for focus categorization including only participants who achieved 6/6 on the headphone screening test (N = 54) and excluding block with repeated stimulus (225 stimuli per condition per subject).

| Effect | Estimate | Std. Error | z-value | p-value |
| --- | --- | --- | --- | --- |
| (Intercept) | -0.568 | 0.099 | -5.742 | < 0.001 |
| Condition | -0.065 | 0.065 | -0.992 | 0.321 |
| F0 Level | 3.385 | 0.303 | 11.177 | < 0.001 |
| Duration Level | 1.598 | 0.157 | 10.208 | < 0.001 |
| Condition x F0 Level | -0.056 | 0.150 | -0.368 | 0.713 |
| Condition x Duration Level | 0.064 | 0.135 | 0.476 | 0.634 |

*Voicing categorization*

Consistent with the results in the main text, there was a significant effect of VOT and F0 on categorization responses (Table S2), and a significant interaction between condition and F0 level. Correlational analyses showed stable individual differences in cue weighting across clear and competing speech conditions (rho = 0.527, p = 0.011). Listeners who strongly weighted a single primary dimension in quiet showed a greater shift towards integrating across multiple dimensions in the presence of competing speech (rho = 0.710, p < 0.001).

**Table S2.** Results of mixed effects model for voicing categorization including only participants who achieved 6/6 on the headphone screening test (N = 23) and excluding block with repeated stimulus (225 stimuli per condition per subject). Note that with this reduced dataset, this model yielded a singular fit suggesting possible overfitting.

| Effect | Estimate | Std. Error | z-value | p-value |
| --- | --- | --- | --- | --- |
| (Intercept) | 0.875 | 0.211 | 4.146 | < 0.001 |
| Condition | -0.573 | 0.148 | -3.879 | < 0.001 |
| VOT Level | 3.661 | 0.322 | 11.366 | < 0.001 |
| F0 Level | 2.572 | 0.249 | 10.348 | < 0.001 |
| Condition x VOT Level | -0.009 | 0.307 | -0.028 | 0.978 |
| Condition x F0 Level | 0.495 | 0.235 | 2.105 | 0.035 |
